# Paternal eNOS deficiency in mice affects glucose homeostasis and liver glycogen in male offspring without inheritance of eNOS deficiency itself

**DOI:** 10.1007/s00125-022-05700-x

**Published:** 2022-04-30

**Authors:** Berthold Hocher, Yong-Ping Lu, Christoph Reichetzeder, Xiaoli Zhang, Oleg Tsuprykov, Jan Rahnenführer, Li Xie, Jian Li, Liang Hu, Bernhard K. Krämer, Ahmed A. Hasan

**Affiliations:** 1 Fifth Department of Medicine (Nephrology/Endocrinology/Rheumatology), University Medical Centre Mannheim, University of Heidelberg, Heidelberg, Germany; 2 Key Laboratory of Study and Discovery of Small Targeted Molecules of Hunan Province, School of Medicine, Hunan Normal University, Changsha, China; 3 Reproductive and Genetic Hospital of CITIC-Xiangya, Changsha, China; 4 Institute of Medical Diagnostics, IMD Berlin, Berlin, Germany; 5 Department of Nephrology, Center of Kidney and Urology, The Seventh Affiliated Hospital, Sun Yat-sen University, Shenzhen, China; 6 Institute of Nutritional Science, University of Potsdam, Nuthetal, Germany; 7 Institute of Pharmacy, Freie Universität Berlin, Berlin, Germany; 8 Institute of Reproductive and Stem Cell Engineering, School of Basic Medical Science, Central South University, Changsha, China

**Keywords:** eNOS, Glucocorticoid receptor, Insulin resistance, Paternal programming, PGC1a

## Abstract

**Aims/hypothesis:**

It was shown that maternal endothelial nitric oxide synthase (eNOS) deficiency causes fatty liver disease and numerically lower fasting glucose in female wild-type offspring, suggesting that parental genetic variants may influence the offspring’s phenotype via epigenetic modifications in the offspring despite the absence of a primary genetic defect. The aim of the current study was to analyse whether paternal eNOS deficiency may cause the same phenotype as seen with maternal eNOS deficiency.

**Methods:**

Heterozygous (+/−) male eNOS (*Nos3*) knockout mice or wild-type male mice were bred with female wild-type mice. The phenotype of wild-type offspring of heterozygous male eNOS knockout mice was compared with offspring from wild-type parents.

**Results:**

Global sperm DNA methylation decreased and sperm microRNA pattern altered substantially. Fasting glucose and liver glycogen storage were increased when analysing wild-type male and female offspring of +/− eNOS fathers. Wild-type male but not female offspring of +/− eNOS fathers had increased fasting insulin and increased insulin after glucose load. Analysing candidate genes for liver fat and carbohydrate metabolism revealed that the expression of genes encoding glucocorticoid receptor (*Gr*; also known as *Nr3c1*) and peroxisome proliferator-activated receptor gamma coactivator 1-alpha (*Pgc1a*; also known as *Ppargc1a*) was increased while DNA methylation of *Gr* exon 1A and *Pgc1a* promoter was decreased in the liver of male wild-type offspring of +/− eNOS fathers. The endocrine pancreas in wild-type offspring was not affected.

**Conclusions/interpretation:**

Our study suggests that paternal genetic defects such as eNOS deficiency may alter the epigenome of the sperm without transmission of the paternal genetic defect itself. In later life wild-type male offspring of +/− eNOS fathers developed increased fasting insulin and increased insulin after glucose load. These effects are associated with increased *Gr* and *Pgc1a* gene expression due to altered methylation of these genes.

**Graphical abstract:**

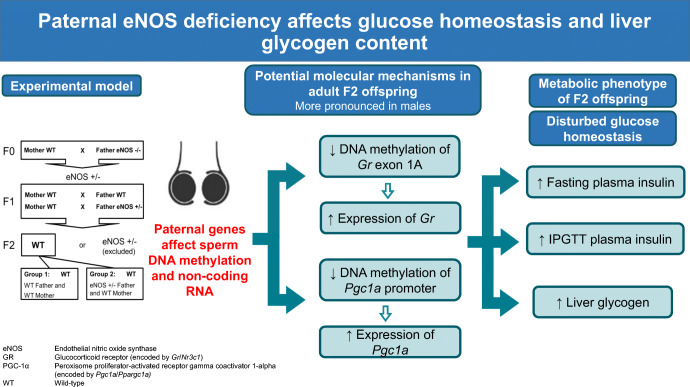

**Supplementary Information:**

The online version contains peer-reviewed but unedited supplementary material available at 10.1007/s00125-022-05700-x.

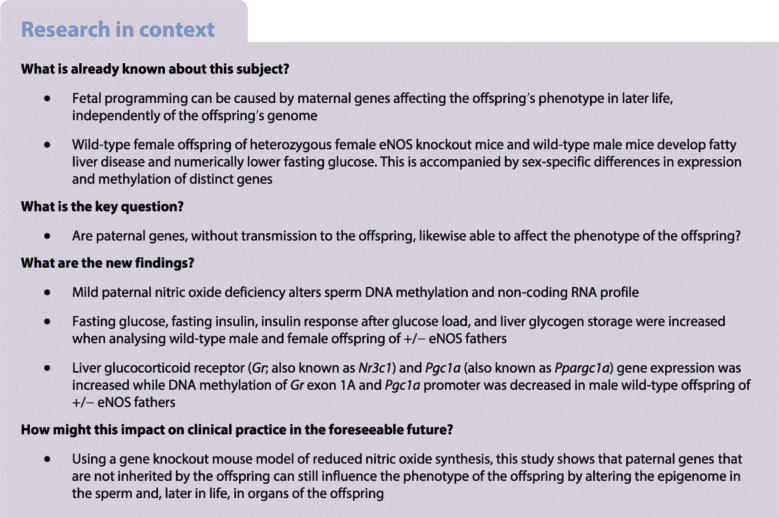



## Introduction

**Fig. 1:**
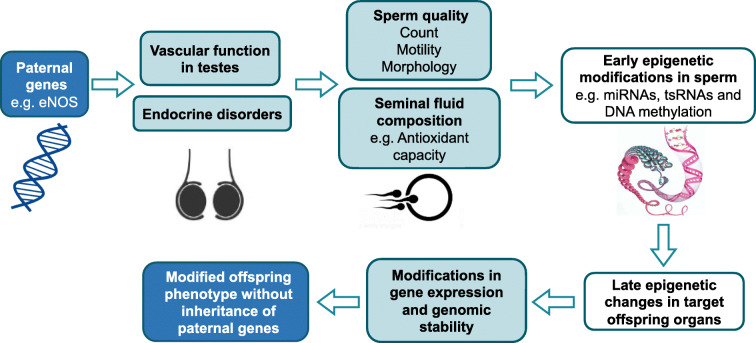
Paternal programming hypothesis. The paternal genetic defects might impact on the offspring phenotype via genomic–epigenomic interactions without inheritance of the defective paternal genes. The paternal genetic changes might affect the endocrine system and vascular function in testes leading to alterations related to sperm quality and seminal fluid composition, which might in turn trigger early epigenetic modifications in sperm, e.g. miRNAs, tRNA-derived small RNAs (tsRNAs) and DNA methylation [18, 20, 36, 37, 64, 65]. These early epigenetic alterations might impact the offspring leading to late epigenetic changes in target offspring organs with modified gene expression and phenotype without transmittance of the paternal genetic change

The ‘fetal origin of diseases’ hypothesis proposes that adulthood diseases originate through adaptation of the fetus to environmental conditions in early life [1]. Another mechanism responsible for programming events might be related to maternal genes affecting the fetal phenotype independently of the offspring’s genome [2–14]. These clinical association studies stimulated the initiation of animal studies to identify the underlying molecular mechanisms. Heterozygous (+/−) female mice in which the *Nos3* gene, encoding endothelial nitric oxide synthase (eNOS), was knocked out and wild-type (WT) female mice were bred with male WT mice. Female offspring with normal *Nos3* gene status but born to heterozygous female eNOS knockout mice develop hepatic steatosis [15], causally demonstrating that maternal genes can epigenetically alter the offspring’s phenotype without inheritance of the gene itself [15]. Paternal environmental factors prior to mating likewise affect the offspring’s phenotype [1, 16]. It was shown that a pre-conceptional paternal high-fat diet results in impaired glucose tolerance in female offspring [17–21]. There are already studies suggesting that paternal genes without transmission to the offspring might likewise affect the offspring’s phenotype [22–24] (Fig. 1).

We have chosen male heterozygous eNOS knockout mice to test this hypothesis, because eNOS plays an important role in the control of testicular vascular function, and hence heterozygous eNOS deficiency in male mice might create an unfavourable testicular microenvironment. We hypothesised that this could influence the offspring’s phenotype. We analysed the impact of paternal nitric oxide (NO) deficiency on epigenetic alterations in sperm. Next, we analysed the phenotype of WT offspring of male heterozygous eNOS knockout mice, followed by analysis of candidate genes (both gene expression and related epigenetic alterations of differentially expressed genes) potentially responsible for the observed phenotype.

## Methods

For detailed methods, please refer to the electronic supplementary material (ESM).

### Breeding protocol and study protocol of eNOS-deficient mice

Male heterozygous mice (C57BL/6 J background) in which the *Nos3* gene encoding eNOS was knocked out [25] were bred with C57BL/6 J female mice and their WT offspring were compared with offspring from healthy male and female C57BL/6 J mice. The breeding procedure is described in more detail in ESM Fig. 1. Study design and experimental protocols were conducted according to the local institutional guidelines for the care and use of laboratory animals and were approved by the animal welfare ethical committee of the state of Berlin.

Male and female offspring were kept for 24 weeks and analysed separately. Body weight, length, abdominal diameter, blood pressure and plasma creatinine were measured and IPGTT was performed. Experimenters were blind to group assignment and outcome assessment for the entire study.

### Effects of NO deficiency on sperm development and epigenetic alterations in the sperm

A total of 30 C57BL/6 J male mice were randomised into three groups and treated with different doses of *N*(γ)-nitro-**l**-arginine methyl ester (l-NAME) for 12 consecutive weeks.

### Sperm total DNA methylation

Mature sperm were isolated from cauda epididymis. Sperm total DNA methylation was performed as described before [26].

### Sperm count and small RNA library construction

Mature sperm were isolated from cauda epididymis of male C57BL/6 J mice and processed for RNA extraction as previously described [27, 28]. Small RNA libraries were constructed. After validation of library quality, sequencing was performed by Illumina HiSeq (Illumina, UK).

### Testicular morphology

Testes were fixed, processed and stained with haematoxylin and eosin, followed by computer-aided image analysis.

### Liver morphology

Livers were fixed, embedded in paraffin and cut into slices. Haematoxylin and eosin staining, Oil Red O staining and immunohistochemistry were performed, followed by computer-aided image analysis.

### Pancreas morphology

Pancreases were fixed, embedded in paraffin and cut into slices. Haematoxylin and eosin staining and immunohistochemistry were performed, followed by computer-aided image analysis.

### Liver glycogen content

Glycogen content was determined using the amyloglucosidase method [29].

### Quantitative real-time PCR

Quantitative real-time RT-PCR was used to determine the relative expression levels of mRNAs as described recently [15]. Sequences of primers used are listed in ESM Table 1.

### Quantification of gene-specific DNA methylation

Quantification of gene-specific DNA methylation was achieved with methylated genomic DNA immunoprecipitation (MeDIP), with minor modifications as described by Weber et al [30].

### Statistics

For the statistical analysis of IPGTT glucose and insulin, two-way ANOVA test followed by Bonferroni post hoc test was conducted. The unpaired Student’s *t* test and Pearson correlation analysis were applied for normally distributed data, while the Mann–Whitney *U* test and Spearman correlation analysis were used for non-normally distributed data. To correct for multiple testing in the gene expression analysis, a false discovery rate (FDR) cut off was set at 0.05 [31, 32]. Statistically significant differences were considered as *p*≤0.05.

## Results

### Effects on sperm under conditions of NO deficiency

**Fig. 2:**
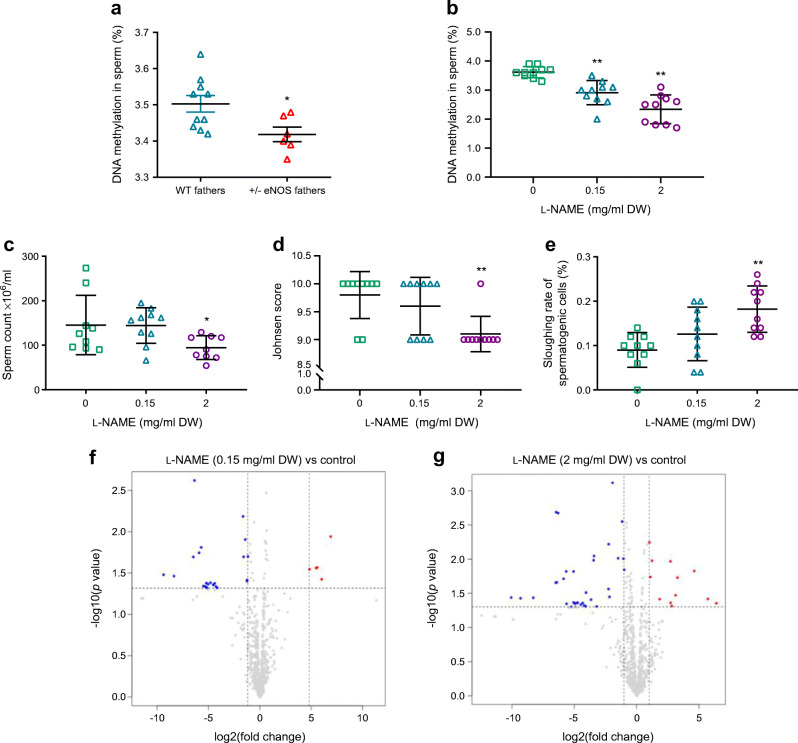
Sperm DNA methylation in WT (*n* = 10) and +/− eNOS fathers (*n* = 6) (**a**), sperm DNA methylation in mice treated with different doses of l-NAME (**b**), sperm count (**c**), determination of testicular morphology: Johnsen score (**d**), sloughing rate of spermatogenic cells (**e**) and volcano plots of differentially expressed miRNAs: low-dose l-NAME (0.15 mg/ml drinking water [DW], *n* = 10) group vs control group (*n* = 10) (**f**) and high-dose l-NAME (2 mg/ml DW, *n* = 10) group vs control group (**g**). *p<0.05 vs WT fathers in (**a**); *p<0.05 and **p<0.01 vs control group in (**b**), (**c**), (**d**) and (**e**)

Sperm total DNA methylation in +/− eNOS fathers was lower than that in WT fathers (Fig. 2a). Mature sperm has a haploid chromosome set. Half of the spermatozoa from heterozygous eNOS knockout mice therefore have an inactivated *Nos3* gene; the remaining half have a normal *Nos3* gene. We therefore treated male WT mice with the identical genetic background as the eNOS knockout mice with l-NAME and then analysed the sperm. This sperm is a well-suited model to analyse effects of reduced eNOS activity in the testes on the maturation of genetically healthy sperm. We have chosen two dosages of l-NAME. The lower dose does not increase blood pressure, whereas the higher dose does. The mice treated with the lower dose can thus be regarded as a model of heterozygous eNOS knockout mice with sperm having only WT *Nos3* genes, because blood pressure is not increased in heterozygous eNOS knockout mice. Total DNA methylation in sperm of mice treated with l-NAME decreased in a dose-dependent manner (Fig. 2b). Sperm count, Johnsen scores and sloughing rate of maturing sperm cells were not altered in mice on low-dose l-NAME (Fig. 2c–e). Twenty-three microRNAs (miRNAs) were downregulated and five miRNAs were upregulated in the low-dose l-NAME group (Fig. 2f). Only six downregulated miRNAs were described previously (miR-615-3p, miR-193a-5p, miR-199b-5p, miR-144-3p, miR-132-3p, miR-8114) (ESM Tables 2–4).

### Birth variables and adult body weight

WT offspring born to +/− eNOS fathers and WT mothers showed no differences in birthweight, length and abdominal diameter when compared with controls (ESM Fig. 2). There was no difference among the groups regarding body weight (ESM Fig. 2, ESM Table 5).

### Blood pressure and kidney function

Neither blood pressure nor kidney function was different in WT offspring born to +/− eNOS fathers and WT mothers as compared with controls (ESM Table 5).

### IPGTT

**Fig. 3:**
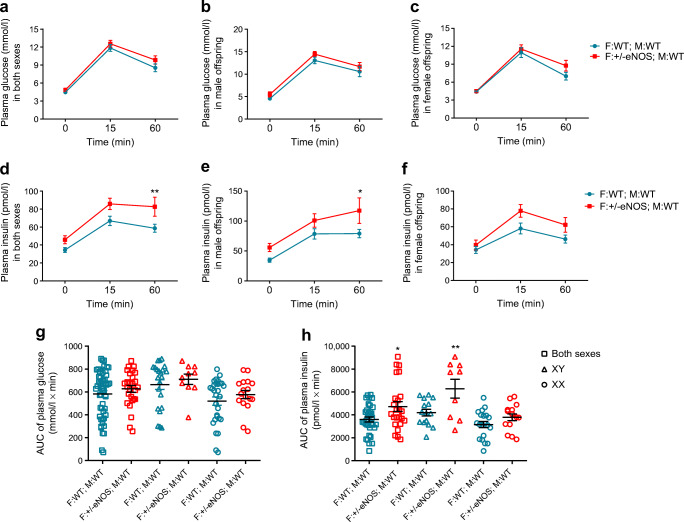
Plasma glucose (**a**–**c**) and insulin concentrations (**d**–**f**) during IPGTT in all (**a**, **d**), male (**b**, **e**) or female (**c**, **f**) offspring; blue circles: father WT/mother WT; red squares: father +/− eNOS/mother WT; AUC for IPGTT plasma glucose (**g**) and IPGTT plasma insulin (**h**) in all (squares) (35 F:WT; M:WT and 24 F:+/−eNOS; M:WT), male (triangles) (15 F:WT; M:WT and 9 F:+/−eNOS; M:WT) or female (circles) (20 F:WT; M:WT and 15 F:+/−eNOS; M:WT) offspring; *p<0.05 and **p<0.01 vs father WT/mother WT. F:+/−eNOS; M:WT, WT offspring of eNOS heterozygous fathers and WT mothers; F:WT; M:WT, WT offspring of WT fathers and WT mothers; XX, female offspring; XY, male offspring

During IPGTT, no differences in glucose concentrations could be observed (Fig. 3). Higher insulin concentrations after an i.p. glucose load, however, were found in animals born to +/− eNOS fathers and WT mothers. Sex-specific analyses showed higher insulin levels in male animals after 60 min of the IPGTT. The analyses of the insulin AUC for offspring from +/− eNOS fathers and WT mothers showed a higher AUC compared with controls. Considering offspring sex revealed that this effect was significant only in male offspring (Fig. 3).

### Fasting plasma glucose and insulin

**Fig. 4:**
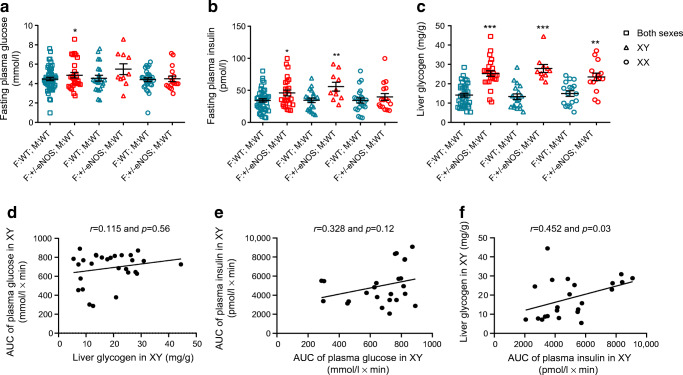
Main phenotypic changes in the offspring: fasting plasma glucose (**a**), fasting plasma insulin (**b**) and liver glycogen (**c**) in all (squares) (35 F:WT; M:WT and 24 F:+/−eNOS; M:WT), male (triangles) (15 F:WT; M:WT and 9 F:+/−eNOS; M:WT) or female (circles) (20 F:WT; M:WT and 15 F:+/−eNOS; M:WT) offspring. Correlation analysis between liver glycogen and AUC of plasma glucose (**d**), AUC of plasma glucose and AUC of plasma insulin (**e**), and AUC of plasma insulin and liver glycogen (**f**) in male offspring. *p<0.05, **p<0.01 and ***p<0.001 vs WT (F:WT; M:WT). F:+/−eNOS; M:WT, WT offspring of eNOS heterozygous fathers and WT mothers; F:WT; M:WT, WT offspring of WT fathers and WT mothers; XX, female offspring; XY, male offspring

WT offspring of +/− eNOS fathers showed higher fasting glucose concentrations. Sex-specific analyses revealed a numerically non-significant elevation of fasting glucose in female and male WT offspring of +/− eNOS fathers (Fig. 4a). Moreover, fasting plasma insulin was significantly higher in male WT offspring of +/− eNOS fathers (Fig. 4b).

### Liver phenotype

Liver weights, liver lobule dimensions, lobular inflammation connective tissue content and hepatic lipid content were similar in all groups (ESM Table 6). Liver glycogen content in both sexes, however, was higher in animals born to +/− eNOS fathers and WT mothers (*p*<0.001) (Fig. 4c). In male offspring, no significant correlation was found between liver glycogen and AUC of plasma glucose, or between AUC of plasma glucose and AUC of plasma insulin (Fig. 4d, e). However, AUC of plasma insulin was positively correlated with liver glycogen (*r* = 0.452, *p*=0.03) (Fig. 4f). For more details see ESM Table 7.

### Liver eNOS/iNOS expression

Liver eNOS (*Nos3*) and inducible nitric oxide synthetase (iNOS; encoded by *Nos2*) expression were comparable in animals born to +/− eNOS fathers (ESM Table 8).

### Pancreas morphology

**Fig. 5:**
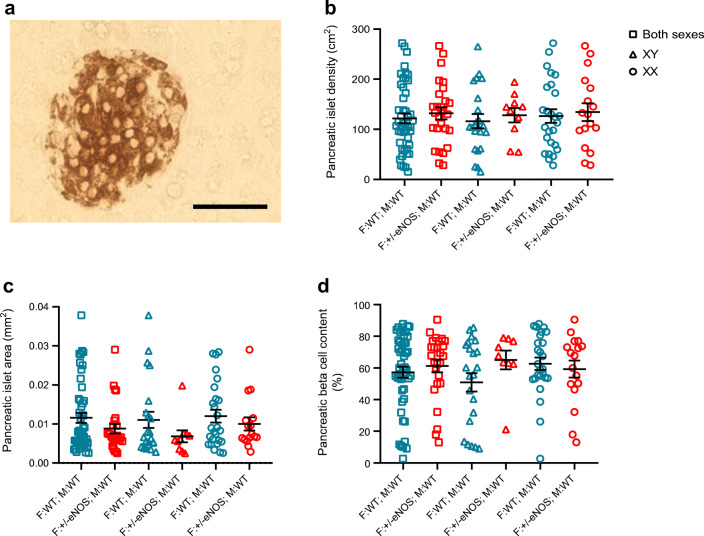
Determination of pancreas morphology: example of insulin stain in beta cells of pancreatic islets of Langerhans (scale bar, 50 μm) (**a**), comparison of islet density (**b**), mean islet area (**c**) and beta cell content (**d**) of islets in all (squares) (35 F:WT; M:WT and 24 F:+/−eNOS; M:WT), male (triangles) (15 F:WT; M:WT and 9 F:+/−eNOS; M:WT) or female (circles) (20 F:WT; M:WT and 15 F:+/−eNOS; M:WT) offspring. F:+/−eNOS; M:WT, WT offspring of eNOS heterozygous fathers and WT mothers; F:WT; M:WT, WT offspring of WT fathers and WT mothers; XX, female offspring; XY, male offspring

Size and density of pancreatic islets of Langerhans and beta cell content of islets were similar in all groups (Fig. 5).

### Quantitative real-time PCR in the liver

WT offspring of both sexes born to +/− eNOS fathers and WT mothers showed an altered expression of genes involved in lipid and carbohydrate metabolism. Seventeen genes were differentially expressed (Table 1). When analysing male offspring born to +/− eNOS fathers and WT mothers (*p*<0.05 and FDR <0.05), 19 genes were differentially expressed (Table 2), with *Gr* (which encodes glucocorticoid receptor [GR]; also known as *Nr3c*1) and *Pgc1a* (which encodes peroxisome proliferator-activated receptor gamma coactivator 1-alpha [PGC-1α]; also known as *Ppargc1a*) showing the lowest *p* values and FDR. Analysing female offspring born to +/− eNOS fathers and WT mothers revealed no differences (Table 3).

**Table 1:** Liver gene expression in both sexes

Gene	F:WT; M:WT (*n* = 20–50)	F:+/−eNOS; M:WT (*n* = 20–26)	*p* value	FDR	Correlation with IPGTT insulin (AUC)		
					Spearman *r*	*p* value	FDR
*Gr* (*Nr3c1*)	1.00 ± 0.09	1.56 ± 0.14	4.9 × 10^−6^	1.3 × 10^–4†^	0.24	0.16	0.54
*Igfbp2*	1.00 ± 0.09	1.28 ± 0.12	1.9 × 10^−4^	2.4 × 10^–3†^	−0.11	0.54	0.81
*Igfbp1*	1.00 ± 0.25	2.15 ± 0.45	2.7 × 10^−4^	2.4 × 10^–3†^	0.26	0.13	0.50
*Fbpase*	1.00 ± 0.07	1.19 ± 0.11	4.7 × 10^−4^	3.2 × 10^–3†^	0.06	0.71	0.80
*Ampk*	1.00 ± 0.09	1.21 ± 0.13	6.6 × 10^−4^	3.6 × 10^–3†^	0.03	0.88	0.88
*Pgc1a*	1.00 ± 0.07	1.56 ± 0.19	8.3 × 10^−4^	3.7 × 10^–3†^	0.18	0.19	0.57
*Cpt1*	1.00 ± 0.08	1.11 ± 0.10	1.7 × 10^−3^	6.4 × 10^–3†^	−0.09	0.60	0.77
*Tfam*	1.00 ± 0.06	1.30 ± 0.08	2.7 × 10^−3^	9.2 × 10^–3†^	0.17	0.20	0.54
*Ppar-Α*	1.00 ± 0.05	1.35 ± 0.12	3.2 × 10^−3^	9.2 × 10^–3†^	0.14	0.29	0.56
*Acc1*	1.00 ± 0.07	1.43 ± 0.13	3.4 × 10^−3^	9.2 × 10^–3†^	0.21	0.12	0.65
*Acsl3*	1.00 ± 0.11	1.64 ± 0.21	4.3 × 10^−3^	1.1 × 10^–2†^	0.23	0.09	0.61
*Acsl4*	1.00 ± 0.07	1.38 ± 0.12	5.3 × 10^−3^	1.2 × 10^–2†^	0.08	0.57	0.77
*Ppar- Γ*	1.00 ± 0.07	1.37 ± 0.13	8.4 × 10^−3^	1.7 × 10^–2†^	0.30	0.02	0.27
*Hsl*	1.00 ± 0.07	0.99 ± 0.10	9.8 × 10^−3^	1.9 × 10^–2†^	−0.27	0.12	0.54
*Gys*	1.00 ± 0.07	1.35 ± 0.13	1.0 × 10^−2^	1.9 × 10^–2†^	0.10	0.44	0.74
*Cdkn1a*	1.00 ± 0.11	2.20 ± 0.59	1.3 × 10^−2^	2.1 × 10^–2†^	0.09	0.53	0.84
*Pdk4*	1.00 ± 0.13	1.79 ± 0.42	2.5 × 10^−2^	4.0 × 10^–2†^	0.33	0.01	0.27
*Nampt*	1.00 ± 0.08	1.32 ± 0.14	3.8 × 10^−2^	5.7 × 10^−2^	0.12	0.37	0.67
*Igfbp3*	1.00 ± 0.12	0.88 ± 0.06	4.6 × 10^−2^	6.6 × 10^−2^	0.07	0.68	0.83
*Srebf1c*	1.00 ± 0.11	0.88 ± 0.09	5.7 × 10^−2^	7.6 × 10^−2^	−0.07	0.70	0.82
*Nrf1*	1.00 ± 0.03	1.11 ± 0.05	5.9 × 10^−2^	7.6 × 10^−2^	0.08	0.55	0.78
*Gck*	1.00 ± 0.06	0.81 ± 0.07	6.5 × 10^−2^	7.8 × 10^−2^	0.17	0.21	0.52
*G6pase*	1.00 ± 0.12	1.34 ± 0.12	6.6 × 10^−2^	7.8 × 10^−2^	0.16	0.24	0.50
*Chrebp*	1.00 ± 0.05	1.17 ± 0.10	9.9 × 10^−2^	1.1 × 10^−1^	0.24	0.06	0.54
*Pck1*	1.00 ± 0.07	1.13 ± 0.11	3.0 × 10^−1^	3.2 × 10^−1^	0.03	0.80	0.86
*Pk-l*	1.00 ± 0.05	1.06 ± 0.12	6.2 × 10^−1^	6.4 × 10^−1^	0.02	0.86	0.89
*Fas*	1.00 ± 0.08	0.94 ± 0.09	6.5 × 10^−1^	6.5 × 10^−1^	0.16	0.22	0.50

Data are given as mean ± SEM and normalised to the reference group (F:WT; M:WT)

^†^Significantly regulated gene with FDR <0.05. They are presented in ascending order according to FDR

F:+/−eNOS; M:WT, WT offspring of eNOS heterozygous fathers and WT mothers; F:WT; M:WT, WT offspring of WT fathers and WT mothers

**Table 2:** Liver gene expression in male offspring

Gene	F:WT; M:WT (*n*= 10–22)	F:+/−eNOS; M:WT (*n*= 10)	*p* value	FDR	Correlation with IPGTT insulin (AUC)		
					Spearman *r*	*p* value	FDR
*Gr* (*Nr3c1*)	1.00 ± 0.15	1.79 ± 0.11	1.80 × 10^−7^	4.85 × 10^–6†^	0.21	0.41	0.74
*Pgc1a* (*Ppargc1a*)	1.00 ± 0.10	1.96 ± 0.15	6.52 × 10^−6^	8.80 × 10^–5†^	0.16	0.45	0.76
*Acsl4*	1.00 ± 0.09	1.84 ± 0.17	4.17 × 10^−5^	3.74 × 10^–4†^	0.15	0.47	0.71
*Acsl3*	1.00 ± 0.15	2.37 ± 0.29	5.54 × 10^−5^	3.74 × 10^–4†^	0.24	0.25	0.68
*Tfam*	1.00 ± 0.08	1.58 ± 0.04	8.99 × 10^−5^	4.85 × 10^–4†^	0.18	0.39	0.75
*Pdk4*	1.00 ± 0.15	2.47 ± 0.37	1.25 × 10^−4^	5.08 × 10^–4†^	0.39	0.06	0.54
*Igfbp1*	1.00 ± 0.35	2.23 ± 0.43	1.32 × 10^−4^	5.08 × 10^–4†^	0.33	0.19	0.73
*Igfbp2*	1.00 ± 0.16	1.47 ± 0.20	1.93 × 10^−4^	5.79 × 10^–4†^	−0.10	0.69	0.81
*Gys*	1.00 ± 0.07	1.68 ± 0.19	1.93 × 10^−4^	5.79 × 10^–4†^	0.01	0.97	0.97
*Fbpase*	1.00 ± 0.10	1.22 ± 0.16	7.60 × 10^−4^	2.05 × 10^–3†^	0.02	0.94	0.98
*Acc1*	1.00 ± 0.12	1.71 ± 0.13	1.75 × 10^−3^	4.22 × 10^–3†^	0.23	0.28	0.69
*Nampt*	1.00 ± 0.12	1.83 ± 0.25	1.87 × 10^−3^	4.22 × 10^–3†^	0.30	0.16	1.08
*Chrebp*	1.00 ± 0.07	1.36 ± 0.06	3.09 × 10^−3^	6.42 × 10^–3†^	0.21	0.33	0.74
*Ampk*	1.00 ± 0.15	1.12 ± 0.14	4.61 × 10^−3^	8.89 × 10^–3†^	−0.13	0.63	0.77
*Pck1*	1.00 ± 0.07	1.44 ± 0.17	8.08 × 10^−3^	1.45 × 10^–2†^	−0.11	0.60	0.77
*Nrf1*	1.00 ± 0.05	1.21 ± 0.05	1.22 × 10^−2^	2.06 × 10^–2†^	0.15	0.49	0.70
*G6pase*	1.00 ± 0.15	1.64 ± 0.14	1.31 × 10^−2^	2.08 × 10^–2†^	0.28	0.18	0.97
*Cdkn1a*	1.00 ± 0.12	3.22 ± 1.37	2.27 × 10^−2^	3.40 × 10^–2†^	0.28	0.18	0.97
*Cpt1*	1.00 ± 0.12	0.92 ± 0.09	2.72 × 10^−2^	3.87 × 10^–2†^	−0.32	0.21	0.71
*Hsl*	1.00 ± 0.10	0.85 ± 0.10	5.59 × 10^−2^	7.55 × 10^−2^	−0.53	0.03	0.41
*Srebf1c*	1.00 ± 0.21	0.90 ± 0.11	6.07 × 10^−2^	7.81 × 10^−2^	0.02	0.93	1.00
*Igfbp3*	1.00 ± 0.19	0.83 ± 0.05	1.01 × 10^−1^	1.24 × 10^−1^	0.19	0.46	0.73
*Ppar-α*	1.00 ± 0.07	1.14 ± 0.09	2.57 × 10^−1^	3.02 × 10^−1^	−0.08	0.72	0.81
*Ppar-γ*	1.00 ± 0.10	1.10 ± 0.10	5.54 × 10^−1^	6.23 × 10^−^^1^	0.57	0.003	0.08
*Pk-l*	1.00 ± 0.10	0.96 ± 0.08	8.00 × 10^−1^	8.64 × 10^−1^	0.12	0.58	0.78
*Fas*	1.00 ± 0.16	1.04 ± 0.18	8.82 × 10^−1^	9.04 × 10^−1^	0.20	0.35	0.73
*Gck*	1.00 ± 0.10	1.02 ± 0.12	9.04 × 10^−1^	9.04 × 10^−1^	0.25	0.24	0.72

Data are given as mean ± SEM and normalised to the reference group (F:WT; M:WT)

^†^Significantly regulated gene with FDR <0.05, and genes are presented in ascending order according to FDR

F:+/−eNOS; M:WT, WT offspring of eNOS heterozygous fathers and WT mothers; F:WT; M:WT, WT offspring of WT fathers and WT mothers

**Table 3:** Liver gene expression in female offspring

Gene	F:WT; M:WT (*n*= 10–28)	F:+/−eNOS; M:WT (*n*= 10–16)	*p* value	FDR	Correlation with IPGTT insulin (AUC)		
					Spearman *r*	*p* value	FDR
*Ppar-α*	1.00 ± 0.08	1.48 ± 0.18	8.45 × 10^−3^	9.94 × 10^−2^	0.41	0.02	0.27
*Ppar-γ*	1.00 ± 0.10	1.54 ± 0.19	9.70 × 10^−3^	9.94 × 10^−2^	0.44	0.01	0.27
*Gck*	1.00 ± 0.08	0.68 ± 0.08	1.10 × 10^−2^	9.94 × 10^−2^	0.02	0.89	1.00
*Cpt1*	1.00 ± 0.12	1.30 ± 0.16	2.36 × 10^−2^	1.52 × 10^−1^	0.29	0.26	1.00
*Ampk*	1.00 ± 0.10	1.30 ± 0.21	3.07 × 10^−2^	1.52 × 10^−1^	0.27	0.30	0.90
*Gr (Nr3c1)*	1.00 ± 0.12	1.32 ± 0.24	3.37 × 10^−2^	1.52 × 10^−1^	0.04	0.89	1.00
*Fbpase*	1.00 ± 0.11	1.16 ± 0.16	5.60 × 10^−2^	1.89 × 10^−1^	0.22	0.39	0.88
*Igfbp1*	1.00 ± 0.37	2.06 ± 0.82	5.60 × 10^−2^	1.89 × 10^−1^	−0.06	0.82	1.00
*Igfbp2*	1.00 ± 0.09	1.09 ± 0.09	6.47 × 10^−2^	1.93 × 10^−1^	−0.31	0.22	1.00
*Hsl*	1.00 ± 0.12	1.12 ± 0.16	7.15 × 10^−2^	1.93 × 10^−1^	0.002	1.00	1.00
*Acc1*	1.00 ± 0.09	1.25 ± 0.19	1.91 × 10^−1^	3.97 × 10^−1^	0.20	0.27	0.91
*Pgc1a*	1.00 ± 0.09	1.32 ± 0.27	1.91 × 10^−1^	3.97 × 10^−1^	0.13	0.46	0.83
*Cdkn1a*	1.00 ± 0.18	1.52 ± 0.32	2.01 × 10^−1^	3.97 × 10^−1^	−0.10	0.57	0.91
*Igfbp3*	1.00 ± 0.14	0.94 ± 0.11	2.06 × 10^−1^	3.97 × 10^−1^	−0.21	0.41	0.79
*Tfam*	1.00 ± 0.08	1.13 ± 0.10	3.28 × 10^−1^	5.67 × 10^−1^	0.22	0.23	1.00
*Srebf1c*	1.00 ± 0.11	0.85 ± 0.15	3.36 × 10^−1^	5.67 × 10^−1^	−0.22	0.39	0.88
*Fas*	1.00 ± 0.08	0.88 ± 0.11	3.64 × 10^−1^	5.77 × 10^−1^	0.16	0.38	0.93
*Gys*	1.00 ± 0.11	1.14 ± 0.15	4.64 × 10^−1^	6.51 × 10^−1^	0.13	0.47	0.79
*Pk-L*	1.00 ± 0.06	1.12 ± 0.19	4.78 × 10^−1^	6.51 × 10^−1^	−0.04	0.85	1.00
*Pdk4*	1.00 ± 0.20	1.37 ± 0.62	4.90 × 10^−1^	6.51 × 10^−1^	0.26	0.15	1.00
*Acsl3*	1.00 ± 0.16	1.18 ± 0.24	5.14 × 10^−1^	6.51 × 10^−1^	0.18	0.32	0.86
*Nrf1*	1.00 ± 0.04	1.05 ± 0.08	5.51 × 10^−1^	6.51 × 10^−1^	0.07	0.71	1.00
*G6pase*	1.00 ± 0.17	1.15 ± 0.15	5.54 × 10^−1^	6.51 × 10^−1^	0.05	0.79	1.00
*Acsl4*	1.00 ± 0.11	1.09 ± 0.12	5.93 × 10^−1^	6.67 × 10^−1^	−0.07	0.69	1.00
*Pck1*	1.00 ± 0.11	0.93 ± 0.13	7.00 × 10^−1^	7.48 × 10^−1^	0.04	0.84	1.00
*Chrebp*	1.00 ± 0.08	1.06 ± 0.15	7.21 × 10^−1^	7.48 × 10^−1^	0.28	0.11	0.99
*Nampt*	1.00 ± 0.11	1.00 ± 0.11	9.83 × 10^−1^	9.83 × 10^−1^	0.001	1.00	1.00

Data are given as mean ± SEM and normalised to the reference group (F:WT; M:WT)

No gene showed significant regulation with FDR <0.05, and genes are presented in ascending order according to FDR

F:+/−eNOS; M:WT, WT offspring of eNOS heterozygous fathers and WT mothers; F:WT; M:WT, WT offspring of WT fathers and WT mothers

### MeDIP methylation analysis in the liver

**Fig. 6:**
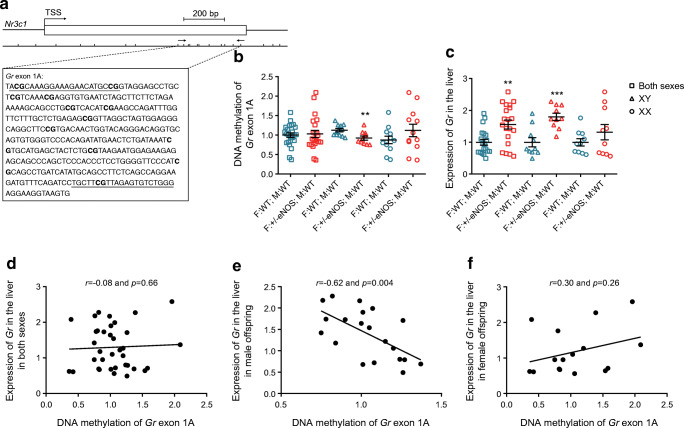
MeDIP methylation analysis of *Gr* (*Nr3c1*) gene exon 1A and *Gr* expression in the liver: (**a**) genomic organisation of *Gr* exon 1A region with putative transcription start site (TSS) and beyond the position of CpG dinucleotides; amplified sequence is shown in the box (primer binding sites are underlined and analysed CpG dinucleotides are in bold letters); (**b**) degree of DNA methylation in amplified region; (**c**) hepatic expression of *Gr* in all (squares) (35 F:WT; M:WT and 24 F:+/−eNOS; M:WT), male (triangles) (15 F:WT; M:WT and 9 F:+/−eNOS; M:WT) or female (circles) (20 F:WT; M:WT and 15 F:+/−eNOS; M:WT) offspring (**p<0.01 and ***p<0.001 vs F:WT; M:WT); and correlation of DNA methylation and gene expression in all (**d**), male (**e**) or female (**f**) offspring. F:+/−eNOS; M:WT, WT offspring of eNOS heterozygous fathers and WT mothers; F:WT; M:WT, WT offspring of WT fathers and WT mothers; XX, female offspring; XY, male offspring

**Fig. 7:**
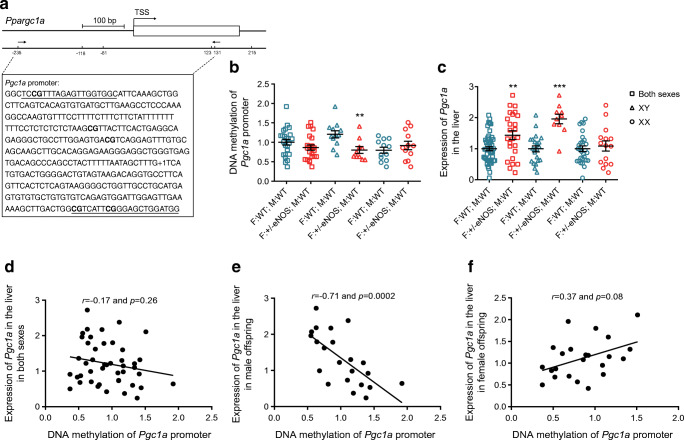
MeDIP methylation analysis of *Pgc1a* (*Ppargc1a*) promoter and *Pgc1a* expression in the liver: (**a**) genomic organisation of *Pgc1a* proximal promoter region with putative transcription start site (TSS/+1) and beyond the position of CpG dinucleotides; amplified sequence is shown in the box (primer binding sites are underlined and analysed CpG dinucleotides are in bold letters); (**b**) degree of DNA methylation in amplified region; (**c**) hepatic expression of *Pgc1a* in all (squares) (35 F:WT; M:WT and 24 F:+/−eNOS; M:WT), male (triangles) (15 F:WT; M:WT and 9 F:+/−eNOS; M:WT) or female (circles) (20 F:WT; M:WT and 15 F:+/−eNOS; M:WT) (**p<0.01 and ***p<0.001 vs F:WT; M:WT); and correlation of DNA methylation and gene expression in all (**d**), male (**e**) or female (**f**) offspring. F:+/−eNOS; M:WT, WT offspring of eNOS heterozygous fathers and WT mothers; F:WT; M:WT, WT offspring of WT fathers and WT mothers; XX, female offspring; XY, male offspring

MeDIP analysis revealed lower *Gr* exon 1A and *Pgc1a* promoter DNA methylation in WT male offspring of eNOS +/− fathers compared with controls (Figs 6, 7). Correlation analysis between the gene expression of liver *Gr* and *Pgc1a* and DNA methylation of *Gr* exon 1A and *Pgc1a* promoter in WT male offspring of eNOS +/− fathers revealed an inverse correlation.

## Discussion

To test the advanced fetal programming hypothesis [2–13] for paternal genes, we used a comparable approach as in our previous study [15] by breeding male heterozygous eNOS knockout mice with female WT mice and comparing the phenotype of their WT offspring with the phenotype of offspring with WT parents. NO deficiency in male mice reduces sperm global DNA methylation and leads to complex changes in non-coding miRNAs in sperm. WT male offspring of +/− eNOS fathers had increased fasting insulin, increased insulin after glucose load and increased liver glycogen content. Since there were no alterations in the endocrine pancreas and liver is the main site of insulin clearance [33, 34], we focused on the liver in our analysis. We found 19 genes differentially expressed in adult male offspring born to +/− eNOS fathers and WT mothers, with *Gr* and *Pgc1a* showing the lowest *p* value and FDR, whereas no differences in gene expression were seen in female offspring. DNA methylation of *Gr* exon 1A and *Pgc1a* promoter in male WT offspring of eNOS +/− fathers was decreased.

### Offspring sex dependency of paternal eNOS deficiency

The phenotype of female WT offspring of +/− eNOS fathers was less pronounced. Only liver glycogen storage was increased. Sex dependency of the offspring’s phenotype is a common phenomenon in fetal programming [1]. One mechanism might be due to offspring sex-dependent transcriptional differences [1, 16]. During preimplantation development, male and female embryos display phenotypic differences that can only be attributed to the transcriptional differences resulting from their different sex chromosomes [35].

### Opposite effects of maternal and paternal eNOS deficiency on glucose homeostasis

The same parental stimulus (heterozygous eNOS deficiency that was not transmitted to the next generation) causes different phenotypes in the offspring. Male WT offspring of +/− eNOS fathers developed a phenotype consisting of increased fasting insulin, increased liver glycogen storage and increased insulin secretion after glucose load. It is remarkable that the effect on fasting glucose seems to go in opposite directions in WT offspring of heterozygous eNOS-deficient mothers (see supplementary Table 2 of our previous publication [15] and Fig. 4). Fasting glucose was numerically lower in WT offspring of +/− eNOS mothers. In contrast, fasting glucose was significantly higher in WT offspring of +/− eNOS fathers compared with controls (Figs 3, 4). Epigenetic alterations were likewise different depending on the parental status of eNOS deficiency. In female WT offspring of +/− eNOS mothers liver fat content correlated significantly with fat storage-inducing transmembrane protein 1 (*Fitm1*) gene expression and *Fitm1* methylation was significantly decreased, whereas DNA methylation of *Gr* exon 1A and *Pgc1a* promoter in male WT offspring of eNOS +/− fathers in comparison with controls was lower while corresponding gene expression was increased.

Taken together, in parental eNOS deficiency the offspring phenotype strongly depends on whether the genetic defect was present in the mother or in the father. The different consequences of the same parental genetic defect (eNOS deficiency) can probably be explained by different impacts of eNOS deficiency for the maturation of the egg or the intrauterine development of the embryo (Figs 1, 2) [1, 16]. Paternal eNOS deficiency affects maturation and development of the sperm and finally alters the epigenome of the sperm, potentially causing long-lasting secondary epigenetic alterations resulting in an adult phenotype characterised by increased fasting insulin, increased insulin after glucose load and increased liver glycogen content. Studies showing that a pre-conceptional paternal high-fat diet results in an impaired glucose tolerance in female offspring due to epigenetic sperm and target organ alterations [17–19, 21] fit well with our findings.

Polymorphisms in the human *NOS3* gene (encoding eNOS) are associated with alterations in the composition of seminal plasma. eNOS deficiency-mediated changes in seminal plasma might thus also be a contributing factor [36, 37]. Maternal eNOS deficiency may affect egg maturation and intrauterine development [1, 15]. In this context, it is of note that parental diabetes has opposite effects on offspring birthweight [38], most likely due to the different effects of paternal and maternal diabetes on spermatogenesis and oocyte/intrauterine development, respectively.

### Can the phenotype in male offspring be explained by an upregulation of GRs?

The hepatic phenotype in WT male offspring of heterozygous eNOS-deficient fathers is in agreement with studies showing that exposure to hepatic GR inhibition lowers glucose in *ob*/*ob* mice [39] and that hepatic GR blockade decreases glucose production and improves insulin resistance [40, 41]. Excess glucocorticoid exposure causes hyperglycaemia and insulin resistance. Our finding of no differences in the glucose response to glucose load in WT male offspring of heterozygous eNOS-deficient fathers vs WT offspring of WT parents but marked differences with respect to insulin levels between WT male offspring of heterozygous eNOS-deficient fathers and controls (Figs 3, 4) suggests that paternal eNOS deficiency causes insulin resistance in the WT male offspring of male heterozygous eNOS knockout mice. In the liver, glucocorticoids increase glycogen storage [42]. We assume that an increased expression of the hepatic GR may have similar consequences. It was reported that GR interacts with insulin degrading enzyme [43]. Since the liver is the primary site for insulin clearance [33, 34], upregulated hepatic GR expression might likewise lead to increased insulin levels. This pathway is androgen-dependent [43]. Hepatic androgen-dependent GR effects on insulin might explain the observed phenotype of increased plasma insulin levels in male WT offspring of male heterozygous eNOS knockout mice.

The human *GR* gene (also known as *NR3C1*) comprises nine exons in which exons 2 to 9 are the protein-encoding region. This gene has a long complex promoter region (the 5′ untranslated region) which is similar to the mouse and rat *Gr* gene [44]. The mouse *Gr* gene has five distinct promoter regions which are 1A, 1B, 1C, 1D and 1E. Exon 1A is found 32 kb upstream from exon 2, and its expression was only detected in tissues with high GR content [45, 46]. Thus, *Gr* exon 1A was selected for DNA methylation analysis and indeed we saw decreased methylation of *Gr* exon 1A and increased *Gr* gene expression in the liver of male WT offspring of eNOS-deficient fathers. The *GR* gene in particular has been shown to be sensitive to early-life environmental conditions, and this effect has been attributed to epigenetic mechanisms [47].

### *PGC1a* methylation and gene expression

DNA methylation of the *PGC1a* gene promoter modulates insulin resistance and is strongly associated with plasma fasting insulin [48, 49]. A study in patients with non-alcoholic fatty liver disease showed that *PGC1a* promoter methylation was inversely correlated with liver *PGC1a* mRNA expression. In addition, *PGC1a* promoter methylation was inversely correlated with HOMA-IR, fasting glucose and insulin. *PGC1a* promoter methylation was also inversely correlated with *PGC1a* promoter methylation [50]. A study done in a rat fetal programming model likewise found an alteration in DNA methylation and transcription of *Pgc1a*. The genetic and epigenetic modifications of *PGC1a* provide a potential mechanism linking early-life nutrition insult to long-term metabolic disease susceptibility [51].

### Pathophysiological and clinical implications

Many studies have indicated that insulin resistance can be caused by fetal programming. Also, paternal factors prior to mating may influence the epigenome of the sperm and hence the adult offspring’s phenotype [1, 16, 52, 53], as was observed in our study. Male heterozygous eNOS knockout mice might be a model of the human endothelial dysfunction sometimes observed in elderly fathers or fathers with hypertension [54–57]. If our findings can be translated to humans, paternal endothelial dysfunction in men might be a risk factor for developing insulin resistance in offspring. Our current study also supports the advanced fetal programming hypothesis as set out in our previous study [15], where we could demonstrate a maternal *Nos3* gene-driven epigenetic alteration of the offspring’s phenotype. Our current study now proposes a non-environmental mechanism of fetal programming driven by altered paternal *Nos3/NOS3* gene function [58, 59] primarily affecting the sperm epigenome and later in life the methylation of offspring target organ genes, resulting in our case in a liver phenotype.

Our current study and the previous study [15] have some general implications:

1. They break with the classical laws of inheritance. The phenotype of WT offspring born to either male or female heterozygous eNOS knockout mice should be identical to offspring from WT parents. However, this was not the case for offspring of either heterozygous eNOS knockout fathers as shown in this study or heterozygous eNOS knockout mothers as shown previously [15].
2. They challenge a key research tool developed to understand gene function: murine transgenic or knockout animal models. Genetically altered animal models may not only reflect causality between a certain genetic alteration and a resulting phenotype. Altered parental genes may additionally induce epigenetic changes affecting the offspring’s phenotype. This notion is supported by human genome-wide association studies [60]. The clinical implications of our study should be further investigated in monogenic inherited diseases such as thalassemia.

### Study limitations

We used inbred mice for our experiments (see also ESM Fig. 1). However, founder fathers for the control group (WT offspring of WT fathers and WT mothers) and fathers for the investigated group (WT offspring of heterozygous eNOS fathers and WT mothers) are different. Thus, additional unknown genetic differences in the fathers used to generate the control group and the investigated group cannot be fully excluded. It is a study limitation that epigenetic changes caused by paternal NO deficiency were analysed at only two time points, in sperm and in the adult animal at the time of characterisation of the adult phenotype. Dynamic epigenetic changes, particularly during fetal development, and their impact on the adult phenotype are important topics of follow-up projects.

We performed IPGTT by measuring glucose and insulin at 0, 15 and 60 min and not for a longer duration on account of animal welfare. However, IPGTT for only 60 min showed differences among the groups and this duration for IPGTT was reported as a suitable approach previously [61–63]. Although group means for insulin AUC comparing WT male offspring born to heterozygous fathers and WT mothers with controls were clearly different (Fig. 3h), it needs to be mentioned that the variation of individual data in the groups was quite high. This might be due to the fact that this variable is calculated based on several measurements. There might be variations in the amount of glucose injected, the time of blood taking after glucose injection, the body weight of the individual animals as well as variation of the insulin ELISA used. All these variables potentiated the variability of insulin AUC. This is for example completely different from reports of organ weight. Here just the variability of the weight measurement method accounts for the variability of the variable. Moreover, we did not analyse fat tissue and muscles. Given the phenotype, this would have been of interest. Furthermore, we screened for differentially expressed genes by using a large but limited list of candidate genes known to be involved in liver fat and carbohydrate metabolism. We did this because the approach was successfully used in our initial study [15].

### Conclusions

This study shows that paternal genes without passing on to the offspring can influence the offspring’s phenotype by altering the epigenome in the sperm and subsequently later in certain organs in adulthood. The same genetic defect in either the father or the mother without transmission to the next generation results in different offspring phenotypes. Our data specifically suggest that heterozygous eNOS deficiency in male mice might cause an unfavourable testicular environment influencing the sperm epigenome. These primary sperm epigenetic alterations may trigger long-lasting epigenetic and subsequent phenotypic alterations in offspring target organs (Fig. 1).

## Supplementary information

ESM (PDF 240 kb)

## Abbreviations

eNOS: Endothelial nitric oxide synthase
FDR: False discovery rate
GR: Glucocorticoid receptor
iNOS: Inducible nitric oxide synthetase
l-NAME: *N*(γ)-nitro-**l**-arginine methyl ester
MeDIP: Methylated genomic DNA immunoprecipitation
miRNA: microRNA
NO: Nitric oxide
PGC-1α: Peroxisome proliferator-activated receptor gamma coactivator 1-alpha
WT: Wild-type
