# Supplementary material for: Paternal eNOS deficiency in mice affects glucose homeostasis and liver glycogen in male offspring without inheritance of eNOS deficiency itself
Source: Diabetologia. 2022 Apr 30;65(7):1222–36. doi: 10.1007/s00125-022-05700-x (PMC9174141; doi:10.1007/s00125-022-05700-x)
Supplement: Supplementary file 1 — (PDF 240 kb) [file 125_2022_5700_MOESM1_ESM.pdf]

Electronic supplementary material (ESM)

ESM methods

1. Breeding protocol and study protocol of eNOS deficient mice

eNOS knockout mice of the C57BL/6J strain and their wild-type (wt) littermate were obtained from *Gödecke et al.* who described the whole procedure of their generation [1]. These animals were bred in-house according to the procedure described in the ESM Fig.1. Study design and experimental protocols were conducted according to the local institutional guidelines for the care and use of laboratory animals and approved by the animal welfare ethical committee of the state of Berlin.

Male and female offspring were kept for 24 weeks and analysed separately. Body weight, length and abdominal diameter of the F2 generation were measured daily until day 13, thereafter body weight daily until day 40 and weekly hereafter until week 20 of the experiment. Blood pressure was measured using the tail cuff method as previously described in week 24 [2]. Plasma creatinine was measured in week 23. Fasting glucose testing was performed in study week 21 and an intraperitoneal glucose tolerance test (IPGTT) in study week 24. Regarding IPGTT, the animals were fasted overnight, injected intraperitoneally with 2mg Glucose/g body weight, then blood samples were collected through the tail vein at 0, 15 and 60 minutes to measure plasma glucose and insulin as described previously [3-5]. Collecting blood samples after longer duration i.e 90 or 120 minutes was not done accounting for animal welfare and to avoid potential loss of animals.

2. Effects of nitric oxide deficiency on sperm development and epigenetic alterations in the sperm

A total of 30 C57BL/6J male mice were randomized into three groups and treated for a consecutive 12 weeks. The control group was given normal drinking water (n =10). The second group received drinking water containing L-NAME (0.15 mg/ml, approximately 15 mg/kg/day; n = 10; Sigma, St Louis, MO, USA). The third group received drinking water containing L-NAME (2 mg/ml, approximately 200 mg/kg/day; n =10). Daily water consumption was estimated individually for every animal 1 week before the experiment. A regular chow diet was used to feed all experimental subjects.

3. Sperm total DNA methylation

Mature sperm was isolated from cauda epididymis. Sperm total DNA methylation was performed as described before [6]. Briefly, DNA was extracted using a QIAamp DNA Mini Kit from Qiagen (Hilden, Germany). The concentration and quality of the RNA-free DNA solution were determined by a NanoDrop ND-1000 spectrophotometer. DNA hydrolysis was carried out using DNA Degradase Plus from Zymo Research (Freiburg, Germany). DNA methylation was assessed by liquid chromatography-electrospray ionization/multi-stage mass spectrometry (LC-ESI/MS/MS) technique as described previously [7].

4. Sperm count and small RNA libraries construction

Mature sperm were isolated from cauda epididymis of male C57BL/6J mice and processed for RNA extraction as previously described [8, 9]. In brief, sperm were released from cauda epididymis into 5ml phosphate - buffered saline (PBS) maintained at 37℃ for 15 min incubation, then 10 ul semen was taken and placed on a MAKLER sperm counting plate and observed by an optical microscope (Olympus BX 53) at ×400 magnification to assess sperm concentration. After incubation, nylon mesh (pore size: 70 mm) was used to filter the suspension. The sperm were then treated with somatic cell lysis buffer (0.1% SDS, 0.5% Triton X in DEPC H2O) for 40 min on ice to eliminate somatic cell contamination, after which the sperm be pelleted by centrifugation at 600g for 5 minutes. after removal of suspension, the sperm pellet was resuspended and washed twice. The sperm pellet was added with TRIzol reagent, homogenated, followed by RNA extraction. Small RNA libraries were constructed according to Small RNA Sample PreKit (Illumina), the small RNA libraries were prepared followed by library quality validation for sequencing.

Deep sequencing, quality control and Small RNA - seq data analysis

For each RNA library, 10 million reads (raw data) were generated by Illumina Hi - Seq. After quality control, small RNA tags were mapped to mouse genome to analyze their distribution and expression on the genome and annotated with miRNA, tRNA, rRNA and other small noncoding RNA from miRBase19, Genbank and Rfam databases using blastn. To analyze differential expression of small RNAs between L-NAME treated and normal mice sperm, miRNA reads were normalized by TPM（transcripts

per million reads. Those miRNAs that had P value smaller than 0.05 and had the fold change of at least 2 were considered as significantly changed miRNAs.

5. testicular morphology

Small tissue samples of testicle were obtained, fixed and processed by routine histological techniques. Tissue sections of 5 μm thickness were stained with hematoxylin–eosin (H&E) and observed under a microscope (Olympus BX53). Sections were evaluated according to the modified Johnsen scoring system as previously described [10, 11]. The sloughing rate of maturing sperm cells is also calculated (at least 50 seminiferous tubules per sample were analyzed) as previously described [12].

6. Liver morphology

Hematoxylin and Eosin Staining was done after washing the livers in PBS buffer, fixation in 4% (w/v) paraformaldehyde in PBS, embedding in paraffin and cutting into 3 μm slices using a Microm HM230 Microtom. For liver slices, the hepatic venules and their adjacent portal fields were identified by sinusoidal connection [13]. 10 lobules of every liver were thus identified using a Zeiss (Oberkochen, Germany) Axiovert 100 microscope (200x) and photographed with a Leica EC3 digital camera using LAS EZ software (Leica, Wetzlar, Germany). Linear lobular dimensions were measured from the centre of the hepatic vein to the centre of three related portal vein branches using ImageJ (version 1.410, NIH shareware). The mean radius of lobules was calculated for each animal. The extent of lobular inflammation was graded as described previously [14]: score 0, no inflammatory foci; score 1, fewer than two foci per × 200 field; score 2, two to four foci per × 200 field; and score 3, more than four foci per × 200 field.

Red Oil Staining was done as described elsewhere [15]. 30 pictures were taken per organ using an Olympus (Shinjuku, JP) BH-2 microscope (400x) and a digital camera CFW-1310C (Scion Corporation, Frederick, MD). The lipid content and the number and size of lipid droplets were quantified with the ImageJ program.

Liver immunohistohemistry: Liver sections were deparaffinized, boiled with sodium citrate buffer (10 mM sodium citrate, 0.05% Tween 20, pH 6.0) with microwave for 10 min for antigen retrieval, and incubated overnight at 4°C with rabbit polyclonal anti-mouse CD68 antibody (ab125047, Abcam, Cambridge, UK) in 4% BSA/PBS. For

immunostaining, an anti-rabbit staining kit (CTS005, R&D Systems, Minneapolis, MN) and peroxidase anti-peroxidase with the streptavidin-biotin system were used. Immunostaining was followed by hematoxylin for nuclear counterstaining. The number of CD68-positive macrophages in the liver was quantified as described previously [16].

7. Pancreas morphology

Hematoxylin and Eosin-stained pictures of whole tissue slide and of every islet of Langerhans were taken using Zeiss Axiovert 100 microscope (25x/200x) and Leica EC3 digital camera. The islets were counted, and the islet area was measured using ImageJ software to calculate the islet density and the mean islet area per slide.

Pancreas Immunohistochemistry: Beta cell content of islets of Langerhans was measured using immunohistological staining of insulin. We used an antibody against insulin (ab7842, abcam, Cambridge, UK) and a secondary antibody (ab6907, abcam) diluted in antibody diluent (Dako, Glostrup, DK) and for visualisation the ABC staining system (sc2023, Santa Cruz Biotechnology, Santa Cruz, CA) according to the manufacturer's instructions. All islets per slide were photographed using an Olympus BH-2 microscope (200x) and CFW-1310C digital camera. Using ImageJ software, the total islet area and the beta cell area was measured (see also Figure 5). Content of β-cells in islets was expressed as the percentage of positively stained area in the total islet area.

8. Liver glycogen content

Glycogen content was determined using the amyloglucosidase method as described before [17]. Briefly, liver was incubated with 1N KOH (95°C, 30 minutes). Glycogen was precipitated using saturated sodium sulfate solution (Na_2SO_4) and 95% (v/v) Ethanol and washed twice in 60% (v/v) Ethanol. Resuspended glycogen was degraded with 0.1% (w/v) Amyloglucosidase (Sigma-Aldrich, St. Louis, MO) in acetate buffer (0.2 M sodiumacetate, 0.46% (v/v) acetic acid, pH 4.8) for 2 hours at 40°C. Glucose concentration was measured colorimetrically using the Glucose (HK) Assay Kit (Sigma-Aldrich). Double measurements were performed, and glycogen content was expressed in relation to liver weight.

9. Quantitative real time PCR

We analysed a list of candidate genes involved in liver fat and carbohydrate metabolism as described recently [18]. We have choosen this list, because we wanted to investigate whether a heterozygous paternal eNOS knockout that is not transmitted to the next generation has the same effect on the offspring's phenotype – a fatty liver phenotype – as we recently described in the offspring of female heterozygous eNOS mice [6]. RNA extraction from liver tissue, reverse transcription PCR and design of specific primer were done like previously described [19]with the exception that primer were obtained from Sigma-Aldrich, Eurofins (Ebersberg, GER) and Biolegio (Nijmegen, NE).

The PCR was performed on a Mx3000P thermal cycler (Stratagene, La Jolla, CA) with Power SYBR Green PCR Master Mix (Applied Biosystems, Foster City, CA), Sensi Mix or SensiFast low ROX kit (Bioline, London, UK) in accordance with instructions for use. All samples were analysed in triplicate. The PCR reaction efficiency has been proofed by linear regression method and the relative quantity of analysed genes was calculated with the ΔΔCt method as described elsewhere [20]. In short, the Ct values of gene of interest were normalized to the geometric mean of the reference genes HPRT, β-Actin and 18S. These values were normalized against the mean value of the reference group. Sequences of used primers are listed in ESM Table 1.

10. Quantification of gene specific DNA methylation

Quantification of gene specific DNA methylation was achieved with immunoprecipitation of methylated genomic DNA (MeDIP), with minor modifications as described by Weber et al. [21]. Briefly, genomic DNA was extracted from liver tissue by proteinase K treatment, RNAse digestion, phenol-chloroform extraction and precipitation with isopropanol. The DNA was sonicated to obtain random fragments between 300 and 1000 bp. 2 µg of the fragmented DNA was denatured for 10 min at 95°C and precipitated over night at 4°C with 10 µg of monoclonal antibody against 5-methylcytosine (Zymo Research) in IP buffer (10 mM sodium phosphate (pH 7.0), 140 mM NaCl, 0.05% Triton X-100). 20 µl of MagnaChip protein G magnetic beads (Millipore) were added and incubated for 2 h at 4°C to capture the antibodies. Magnetic beads were washed two times with IP buffer and treated with proteinase K for 3 h at 50°C. The methylated DNA was recovered by phenol-chloroform extraction and ethanol precipitation.

For the analysis of GR exon 1A and PGC1a promoter, specific primers were created (ESM Table 1). To calculate the proportion of methylated DNA in specific target sequences the content in enriched methylated DNA and input DNA were compared and normalized against the mean value of the reference group.

11. Statistics

A formal power calculation to plan group size was not possible due to two reasons first the litter size of wildtype offspring cannot be exactly planned a priori. Second, we did not know the resulting phenotype of wildtype offspring from heterozygous eNOS knockout fathers. Thus, there was no clear assumptions about the effect size and variation that we might expect. Given that, we decided that we needed to have a least 10 animals per group and planed our experiments based on this assumption. This approach to plan the study was approved by the responsible animal welfare committee.

Statistical analysis was done using GraphPad Prism 6 software (GraphPad, La Jolla, CA). All values are presented as mean ± SEM. For the statistical analysis of IPGTT glucose and insulin, two-way analysis of variance (2-way ANOVA) test followed by Bonferroni post-hoc test was conducted. The unpaired Student’s t-test and Pearson correlation analysis were applied for normally distributed data, while the Mann-Whitney U test and Spearman correlation analysis were used for non-normally distributed data. The data were non-normally distributed; thus the Mann-Whitney-U test and Spearman’s rank correlation were applied. To correct for multiple testing in the gene expression analysis, a false discovery rate (FDR) cut off was set at 0.05 (observation of no more than 5% false positives) [22, 23]. Statistically significant differences were considered as $p \leqslant 0.05$.

ESM Table 1: Primers used for real time PCR and MeDIP

Target Gene	Primer Pair	Length of amplification product (bp)
18S	fw 5‘CGGCTACCACATCCAAGGAA’ 3<br>rev 5‘GCTGGAATTACCGCGGCT ’ 3	187
Acc1 (Acaca, ENSMUSG00000020532)	fw 5’ TTTCACATGAGATCCAGCATG ‘3<br>rev 5’ GCCACAGTGAAATCTCGTTG ‘3	92
β-Actin (Actb, ENSMUSG00000029580)	fw 5' GATATCGCTGCGCTGGTC '3<br>rev 5' CATCACACCCTGGTGCCTA '3	123
Acsl3 (ENSMUSG00000032883)	fw 5’ CTGTTCCGGAAATCATGGA ‘3<br>rev 5’ GAAAACAAAGCGGTCACACA ‘3	158
Acsl4 (ENSMUSG00000031278)	fw 5’ CCAGAAAACTTGAGCGTTCC ‘3<br>rev 5’ TGTCCTTCGGTCCTAGTCCA ‘3	172
AMPK-α2 (Prkaa2, ENSMUSG00000028518)	fw 5‘AAAGACATACGAGAACATGAATGG’3<br>rev 5‘CTTCACAGCCTCATCGTCAA’3	105
Cdkn1a (ENSMUSG00000023067)	fw 5’ CAGACCAGCCTGACAGATTTC ‘3<br>rev 5’ GCAGGCAGCGTATATACAGGA ‘3	204
CHREBP (Mlxipl, ENSMUSG00000005373)	fw 5‘ GAAGATGCTTATGTTGGCAATG ’3<br>rev 5‘ GGCGGTAATTGGTGAAGAAA ’3	109
CPT1a (ENSMUSG00000024900)	fw 5‘CGCACATTACAAGGACATGG’3<br>rev 5‘TCTGCTCTGCCGTTGTTGT’3	158
eNOS (Nos3, ENSMUSG00000028978)	fw 5‘ GGGAAAGCTGCAGGTATTTG ’3	111

	rev 5‘ GCTGAACGAAGATTGCCTCT '3	
FAS (Fasn, ENSMUSG00000025153)	fw 5‘ GGATTCGGTGTATCCTGCTG ’3<br>rev 5‘ TGGGCTTGTCCTGCTCTAAC ’3	171
Fbp1 (FBPase)(ENSMUSG00000069805)	fw 5' ATCAAAGCCATCTCGTCTGC '3<br>rev 5' ATTTGCCCCTCTTCTCAGGT '3	217
G6Pase (G6pc, ENSMUSG00000078650)	fw 5' GACTGTGGGCATCAATCTCC '3<br>rev 5' TCACAGGTGACAGGGAACTG '3	165
Gck (ENSMUSG00000041798)	fw 5' AAGTCCCACGATGTTGTTCC '3<br>rev 5' CTTCCCTGTAAGGCACGAAG '3	100
GR (Nr3c1, ENSMUSG00000024431)	fw 5' ACACGTCAGCACCCCATAAT '3<br>rev 5' AGGCCGCTCAGTGTTTTCTA '3	160
Gys2 (ENSMUSG00000030244)	fw 5’ GAGGAAGCCTGATGTAGTGACTC ‘3<br>rev 5’ TCCAGATGACCATAGAAATGACC ‘3	138
Hprt (ENSMUSG00000025630)	fw 5'CAGGCCAGACTTTGTTGGAT '3<br>rev 5' TTGCGCTCATCTTAGGCTTT '3	147
HSL (Lipe, ENSMUSG00000003123)	fw 5‘ACCTGCTTGGTTCAACTGGA’3<br>rev 5‘CTGGCACCCTCACTCCATAG’3	111
IGF-BP 1 (Igfbp1, ENSMUSG00000020429)	fw 5' CAGCATGAAGAGGCAAAGG '3<br>rev 5' CTATAGGTGCTGATGGCGTTC '3	152

IGF-BP 2 (Igfbp2, ENSMUSG00000039323)	fw 5' AGGTCCTGGAGCGGATCT '3<br>rev 5' CATCTTGCACTGCTTAAGGTTG '3	125
IGF-BP 3 (Igfbp3, ENSMUSG00000020427)	fw 5' TGCTCCAGGAAACATCAGTG '3<br>rev 5' GGAGTGGATGGAACTTGGAA '3	110
iNOS (Nos2, ENSMUSG00000020826)	fw 5‘ TGACACACAGCGCTACAACA ’3<br>rev 5‘ CCATGATGGTCACATTCTGC ' 3	152
Nampt (ENSMUSG00000020572)	fw 5’ CACCGACTCGTACAAGGTTACTC ‘3<br>rev 5’ TTTCACGGCATTCAAAGTAGG ‘3	83
Nrf1 (ENSMUSG00000058440)	fw 5’ TCATCTCGTACCATCACAGACC ‘3<br>rev 5’ TTTGTTCCACCTCTCCATCAG ‘3	182
Pdk4 (ENSMUSG00000019577)	fw 5’ CTCTTCAAGAATGCCATGAGG ‘3<br>rev 5’ TCGGTCAGAAATCTTGATTGTAAG ‘3	120
PEPCK (Pck1, ENSMUSG00000027513)	fw 5' ATACATGGTGCGGCCTTTC '3<br>rev 5' GACAACTGTTGGCTGGCTCT '3	204
PK-L (Pklr, ENSMUSG00000041237)	fw 5’AGTATGGAAGGGCCAGCA ‘3<br>rev 5’AGAGGTGTTCCAGGAAGGTG ‘3	130
PGC1a (Ppargc1a, ENSMUSG00000029167)	fw 5‘AGTCACCAAATGACCCCAAG’ 3<br>rev 5‘GGAGTTGTGGGAGGAGTTAGG’ 3	106
PPARγ (Pparg, ENSMUSG00000000440)	fw 5‘ CAGGCCTCATGAAGAACCTT’ 3<br>rev 5‘ GGATCCGGCAGTTAAGATCA’ 3	176

PPARα (Ppara, ENSMUSG00000022383)	fw 5’ TCTGGAAGCTTTGGTTTTGC '3<br>rev 5‘ TTCGACACTCGATGTTCAGG '3	176
SREBF1c (Srebf1, ENSMUSG00000020538)	fw 5‘CTGTCGGGGTAGCGTCTG’3<br>rev 5‘CGGGAAGTCACTGTCTTGGT’3	112
Tfam (ENSMUSG00000003923)	fw 5’ ACACCCAGATGCAAAACTTTC ‘3<br>rev 5’ CTTTGTATGCTTTCCACTCAGC ‘3	122
PGC1a promoter (5 CpG)	fw 5’ TCCGGTTTAGAGTTGGTGGC ‘3<br>rev 5’ CCATCCAGCTCCCGAATGAC ‘3	380
GR exon 1A (11 CpG)	fw 5’ ACGCAAAGGAAAGAACATGCC ‘3<br>rev 5’ CCCAGACACTCTAACGAAGCA ‘3	349

ESM Table 2. Differentially expressed miRNAs between low-dose L-NAME (0.15mg/ml DW) group and control group

miRNA	logFC	P Value
**Down-regulated miRNA**		
mmu-miR-615-3p	-1.188126408	0.019959695
mmu-miR-193a-5p	-1.27414962	0.038590132
mmu-miR-199b-5p	-1.275405992	0.039922001
mmu-miR-144-3p	-1.45308533	0.01248127
mmu-miR-132-3p	-1.610251375	0.020140258
mmu-miR-8114	-1.661256834	0.006551138
novel_184	-4.18149069	0.048205786
novel_187	-4.301847207	0.046457366
novel_158	-4.427062846	0.04252077
novel_189	-4.572081065	0.044273836
novel_145	-4.831490776	0.041621203
novel_141	-5.059573644	0.043502905
novel_228	-5.152814224	0.048448563
novel_137	-5.223232961	0.047526908
novel_170	-5.235373352	0.042099932
novel_258	-5.341004355	0.046318068
novel_238	-5.52641261	0.04578849
novel_147	-5.737488916	0.015507201
novel_248	-5.947660942	0.018018269
novel_156	-6.393873119	0.002402113
novel_152	-6.502969632	0.020218211
novel_139	-8.381870524	0.034513278
novel_133	-9.400424176	0.033183276
**Up-regulated miRNA**		
novel_66	4.793368	0.028573
novel_58	5.434276	0.027511

novel_29	5.556467	0.027116
novel_77	5.975698	0.037912
novel_31	6.866177	0.011476

ESM Table 3. Differentially expressed miRNAs between high-dose L-NAME (2mg/ml DW) group and control group

miRNA	logFC	P Value
**Down-regulated miRNAs**		
mmu-miR-1843a-5p	-1.022555086	0.014345528
mmu-miR-503-5p	-1.085633053	0.009853503
novel_33	-1.192791837	0.002824878
mmu-miR-7230-3p	-1.515984779	0.009723282
novel_28	-1.955217119	0.000764771
mmu-miR-5099	-2.205040054	0.035678986
mmu-miR-497a-3p	-2.269705466	0.006030488
novel_84	-2.280232855	0.027527084
mmu-miR-181d-3p	-3.226742279	0.049292565
mmu-miR-7015-3p	-3.4442631	0.009004629
mmu-miR-802-5p	-3.477390754	0.010386511
mmu-miR-1934-5p	-3.687785393	0.039205574
mmu-miR-128-1-5p	-4.083115839	0.030951033
novel_184	-4.109186325	0.049069552
novel_187	-4.230645211	0.047614594
novel_158	-4.352196753	0.043753659
novel_189	-4.498326021	0.046217523
novel_145	-4.757665544	0.043398889
novel_88	-4.772178861	0.043502864
novel_79	-4.923206711	0.04426357
novel_120	-4.947664744	0.049891703
novel_141	-4.991829808	0.044516861
novel_114	-5.085712365	0.042926355
novel_21	-5.086738329	0.015148642
novel_258	-5.271151919	0.049804242
novel_42	-5.63758224	0.044847908
novel_147	-5.670669564	0.015130277

novel_248	-5.886025363	0.019359849
novel_156	-6.321290758	0.002119395
novel_152	-6.43052072	0.022028445
novel_78	-6.483633127	0.002054752
novel_107	-6.510814481	0.022250835
novel_139	-8.312733009	0.036698075
novel_133	-9.333723567	0.037405496
novel_121	-10.0821985	0.036392588
**Up-regulated miRNAs**		
mmu-miR-320-3p	1.000850931	0.005684666
mmu-miR-365-3p	1.072714256	0.018210289
mmu-miR-146a-5p	1.207131152	0.010556529
mmu-miR-292a-5p	1.818042926	0.03852769
mmu-miR-219c-5p	2.686836576	0.010766331
mmu-miR-6970-5p	2.695362472	0.043526378
mmu-miR-218-1-3p	2.787253559	0.048315625
mmu-miR-7219-3p	3.09932527	0.033766784
mmu-miR-3060-3p	3.247223663	0.018640336
novel_66	4.592328379	0.014903218
novel_215	5.690671331	0.038178473
novel_77	6.36285523	0.044196678

ESM Table 4: Described functions of already decribed miRNAs identifyied in mouse sperm after low dose L-NAME treatment mimiking paternal heterozygous deficiency.

miRNA	Main findings	Reference
mmu-miR-615-3p	microRNA mmumiR-615-3p is reduced under conditions of endoplasmic reticulum (ER) stress, wherein it regulates the expression of C/EBP homologous protein (CHOP) and determines cellular sensitivity to cell death.	[24]
	CircZNF609 is involved in the pathogenesis of focal segmental glomerulosclerosis by sponging miR-615-5p	[25]
mmu-miR-193a-5p	miR-193a alleviates diabetic neuropathic pain in a mouse model through the inhibition of HMGB1 expression	[26]
	miR-193a/b-3p overexpression attenuates liver fibrosis through suppressing the proliferation and activation of HSCs.	[27]
	mmu-miR-193 influenced embryo implantation by regulating growth factor receptor-bound protein 7 expression.	[28]
mmu-miR-199b-5p	miR-199b-5p is an important regulator in medullary TEC proliferation through targeting Fzd6 to activate Wnt signaling and cell cycle signaling.	[29]
	miR-199b as a regulator of the phenotypic switch during vascular cell differentiation derived from iPS cells by regulating critical signaling angiogenic responses	[30]
	miR-199b is a direct calcineurin/NFAT target gene that increases in expression in mouse and human heart failure	[31]
mmu-miR-144-3p	miR-144 is involved in extracellular matrix remodeling post MI and its loss leads to increased myocardial fibrosis and impaired functional recovery.	[32]
	Downregulation of microRNA-144 inhibits proliferation and promotes the apoptosis of myelodysplastic syndrome cells through the activation of the AKAP12-dependent ERK1/2 signaling pathway	[33]

	miR-144 maybe a potential regulator of the development of atherosclerosis via changes in vimentin signaling.	[34]
	Circulating exosomal miR-144-3p inhibits the mobilization of endothelial progenitor cells post myocardial infarction via regulating the MMP9 pathway.	[35]
mmu-miR-132-3p	miR-132-3p priming enhances the effects of mesenchymal stromal cell-derived exosomes on ameliorating brain ischemic injury	[36]
	Targeted silencing of miRNA-132-3p expression rescues disuse osteopenia by promoting mesenchymal stem cell osteogenic differentiation and osteogenesis in mice	[37]
	Brown adipocyte-derived exosomal miR-132-3p suppress hepatic Srebf1 expression and thereby attenuate expression of lipogenic genes	[38]
	MiR-132 controls pancreatic beta cell proliferation and survival through Pten/Akt/Foxo3 signaling	[39]
mmu-miR-8114		No functional related literature found

ESM Table 5: Body weight, organ weights, systolic blood pressure and plasma creatinine

Variable	Both sexes		XY		XX	
	F:WT; M:WT (n=45-50)	F:+/-eNOS; M:WT (n=26-27)	F:WT; M:WT (n=20-22)	F:+/-eNOS; M:WT (n=10)	F:WT; M:WT (n=25-28)	F:+/-eNOS; M:WT (n=16-17)
Final body weight (g)	27.25±0.99	27.07±1.11	34.18±0.89	32.80±1.28	21.80±0.42	23.70±0.83 **
Relative Liver Weight (% of body weight)	4.31±0.06	4.26±0.08	4.56±0.07	4.44±0.10	4.11±0.08	4.16±0.11
Relative Kidney weight (% of body weight)	0.59±0.01	0.61±0.01	0.61±0.02	0.64±0.02	0.57±0.01	0.59±0.02
Relative Heart weight (% of body weight)	0.51±0.01	0.49±0.01	0.50±0.01	0.49±0.02	0.51±0.01	0.49±0.02
Systolic blood pressure (mmHg)	102.70±2.43	106.10± 2.16	95.65± 3.22	99.04± 2.14	108.30± 3.20	110.30± 2.76
Plasma creatinine (µmol/l)	52.16±0.02	49.50±0.03	48.62±0.02	46.85±0.03	55.69±0.03	50.39±0.04

XX: female offspring; XY: male offspring. Data are given as mean±SEM and **: p<0.01 vs. father wt/mother wt

ESM Table 6: Histological findings in the liver

Variable	Both sexes		XY		XX	
	F:WT; M:WT (n=48)	F:+/-eNOS; M:WT (n=26-27)	F:WT; M:WT (n=22)	F:+/-eNOS; M:WT (n=9-10)	F:WT; M:WT (n=26)	F:+/-eNOS; M:WT (n=17)
Liver Lobular Dimension (mm)	0.07±0.001	0.07±0.001	0.07±0.001	0.07±0.002	0.07±0.003	0.07±0.002
Liver Connective Tissue Content (% area)	0.16±0.02	0.17±0.03	0.12±0.03	0.11±0.04	0.18±0.03	0.20±0.04
Liver Fat Content (% area)	0.73±0.16	1.09±0.34	0.87±0.32	0.20±0.06	0.61±0.10	1.67±0.51
Liver Lipid Droplet Density (droplets/mm^2)	2701.0± 411.2	3066.1±689.0	2537.7±779.2	4980.7±255.5	2850.8±352.1	4406.6±969.9
Liver Lobular Inflammation (score)	0.31±0.13	0.33±0.14	0.29±0.18	0.33±0.21	0.33±0.21	0.33±0.21
Number of CD68-Positive Immune Cells (score) in the liver	0.85±0.10	0.92±0.08	0.86±0.14	1.00±0.00	0.83±0.17	0.83±0.17

XX: female offspring; XY: male offspring. Data are given as mean±SEM

ESM Table 7: Correlation among plasma glucose, plasma glucose and liver glycogen.

Parameter	Liver glycogen	IPGTT plasma glucose (0 min)	IPGTT plasma glucose (15 min)	IPGTT plasma glucose (60 min)	IPGTT plasma glucose (AUC)
**Both sexes**					
Liver glycogen	1.0	0.128	-0.017	0.161	0.087
IPGTT plasma insulin (0 min)	0.305*	-0.054	0.031	0.054	0.001
IPGTT plasma insulin (15 min)	0.2	-0.008	0.425**	0.399**	0.414**
IPGTT plasma insulin (60 min)	0.107	0.038	0.449**	0.536**	0.545**
IPGTT plasma insulin (AUC)	0.276	0.016	0.435**	0.449**	0.426**
XX					
Liver glycogen	1.0	-0.004	-0.183	0.143	-0.007
IPGTT plasma insulin (0 min)	0.201	-0.148	-0.033	0.330*	0.074
IPGTT plasma insulin (15 min)	0.114	-0.129	0.508**	0.428**	0.464**
IPGTT plasma insulin (60 min)	-0.059	0.105	0.354*	0.619**	0.504**
IPGTT plasma insulin (AUC)	0.183	0.07	0.396*	0.594**	0.430*
XY					
Liver glycogen	1.0	0.24	0.22	0.076	0.115
IPGTT plasma insulin (0 min)	0.34	0.04	0.135	-0.293	-0.114
IPGTT plasma insulin (15 min)	.419*	0.124	0.266	0.196	0.214
IPGTT plasma insulin (60 min)	0.337	-0.069	0.440*	0.254	0.391*
IPGTT plasma insulin (AUC)	0.452*	-0.035	0.473*	0.208	0.328

XX: female offspring; XY: male offspring. *: $p<0.05$, **: $p<0.01$.

ESM Table 8: Liver NOS expression, real time PCR quantification

Variable	Both sexes		XY		XX	
	F:WT; M:WT (n=20)	F: +/- eNOS; M:WT (n=20)	F:WT; M:WT (n=10)	F: +/- eNOS; M:WT (n=10)	F:WT; M:WT (n=10)	F: +/- eNOS; M:WT (n=10)
eNOS (Nos3)	1.00 ± 0.06	1.03 ± 0.08	1.00 ± 0.07	1.04 ± 0.07	1.00 ± 0.11	1.02 ± 0.15
iNOS (Nos2)	1.00 ± 0.13	0.92 ± 0.12	1.00 ± 0.19	0.84 ± 0.10	1.00 ± 0.19	1.00 ± 0.22

XX: female offspring; XY: male offspring. Data are given as mean±SEM

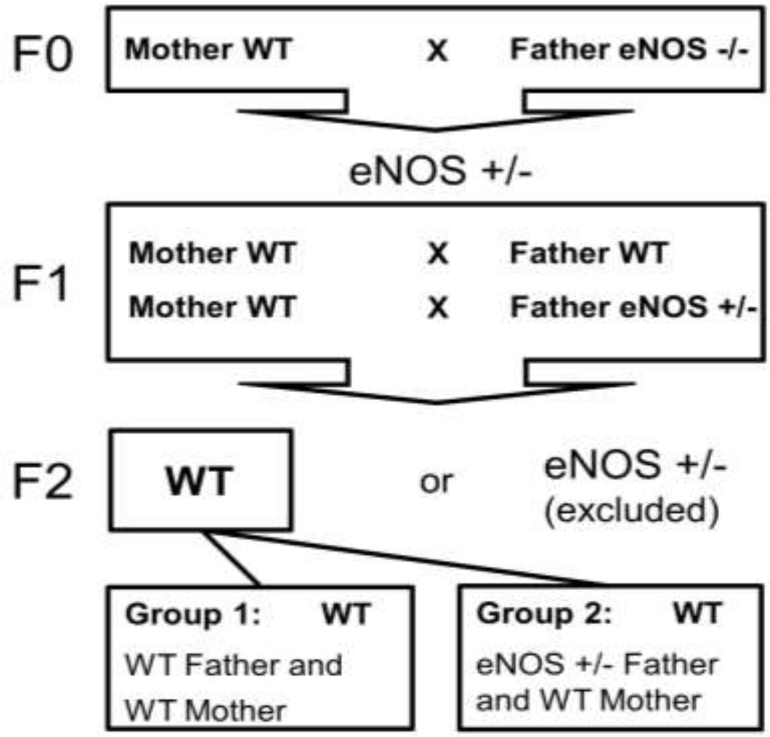

ESM Fig.1:

eNOS knockout mice of the C57BL/6J strain and their wild-type (wt) littermate were used. Female wt mice were cross-bred with homozygous male eNOS knockout mice. The resulting male heterozygous eNOS knockout ($eNOS^{+/-}$) mice were then again crossed with female wt mice. Only wt offspring of this breeding procedure (F2 generation) entered the study. These mice were compared to wt mice resulting from crossing male wt and female wt mice. Heterozygous animals used for breeding of the F2 generation were all derived from different dams i.e siblings were not used.

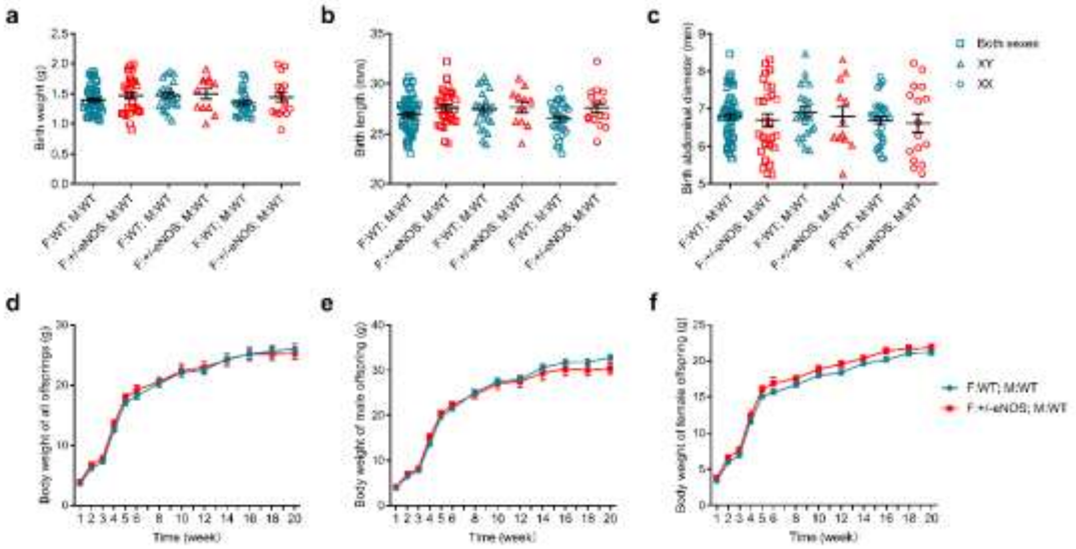

ESM Fig.2:

Birth weight, length and abdominal diameter as well as the change in body weight over the time

XX: female offspring; XY: male offspring. F:WT;M:WT : wildtype offspring of wildtype fathers and wildtype mothers and F:+/-eNOS;M:WT : wildtype offspring of eNOS heterozygous fathers and wildtype mothers

ESM References:

[1] Godecke A, Decking UK, Ding Z, et al. (1998) Coronary hemodynamics in endothelial NO synthase knockout mice. Circ Res 82(2): 186-194. http://doi.org/10.1161/01.res.82.2.186

[2] Quaschning T, Voss F, Relle K, et al. (2007) Lack of endothelial nitric oxide synthase promotes endothelin-induced hypertension: lessons from endothelin-1 transgenic/endothelial nitric oxide synthase knockout mice. J Am Soc Nephrol 18(3): 730-740. http://doi.org/10.1681/ASN.2006050541

[3] Wang Q, Chen K, Liu R, et al. (2010) Novel GLP-1 fusion chimera as potent long acting GLP-1 receptor agonist. PLoS One 5(9): e12734. http://doi.org/10.1371/journal.pone.0012734

[4] Kim BJ, Zhou J, Martin B, et al. (2010) Transferrin fusion technology: a novel approach to prolonging biological half-life of insulinotropic peptides. J Pharmacol Exp Ther 334(3): 682-692. http://doi.org/10.1124/jpet.110.166470

[5] Du X, Kosinski JR, Lao J, et al. (2012) Differential effects of oxyntomodulin and GLP-1 on glucose metabolism. Am J Physiol Endocrinol Metab 303(2): E265-271. http://doi.org/10.1152/ajpendo.00142.2012

[6] Reichetzeder C, Dwi Putra SE, Pfab T, et al. (2016) Increased global placental DNA methylation levels are associated with gestational diabetes. Clin Epigenetics 8: 82. http://doi.org/10.1186/s13148-016-0247-9

[7] Dwi Putra SE, Neuber C, Reichetzeder C, Hocher B, Kleuser B (2014) Analysis of genomic DNA methylation levels in human placenta using liquid chromatography-electrospray ionization tandem mass spectrometry. Cell Physiol Biochem 33(4): 945-952. http://doi.org/10.1159/000358666

[8] Peng H, Shi J, Zhang Y, et al. (2012) A novel class of tRNA-derived small RNAs extremely enriched in mature mouse sperm. Cell Res 22(11): 1609-1612. http://doi.org/10.1038/cr.2012.141

[9] Chen Q, Yan M, Cao Z, et al. (2016) Sperm tsRNAs contribute to intergenerational inheritance of an acquired metabolic disorder. Science 351(6271): 397-400. http://doi.org/10.1126/science.aad7977

[10] Johnsen SG (1970) Testicular biopsy score count--a method for registration of spermatogenesis in human testes: normal values and results in 335 hypogonadal males. Hormones 1(1): 2-25. http://doi.org/10.1159/000178170

[11] Liu CY, Chang TC, Lin SH, Wu ST, Cha TL, Tsao CW (2020) Metformin Ameliorates Testicular Function and Spermatogenesis in Male Mice with High-Fat and High-Cholesterol Diet-Induced Obesity. Nutrients 12(7). http://doi.org/10.3390/nu12071932

[12] Dhakal HP, Coleman J, Przybycin CG (2019) A Novel Dual Immunostain to Characterize Sloughed Cells in Testicular Biopsies for Infertility. Am J Surg Pathol 43(8): 1123-1128. http://doi.org/10.1097/PAS.0000000000001281

[13] Burns SP, Desai M, Cohen RD, et al. (1997) Gluconeogenesis, glucose handling, and structural changes in livers of the adult offspring of rats partially deprived of protein during pregnancy and lactation. J Clin Invest 100(7): 1768-1774. http://doi.org/10.1172/JCI119703

[14] Kim SW, Hur W, Li TZ, et al. (2014) Oleuropein prevents the progression of steatohepatitis to hepatic fibrosis induced by a high-fat diet in mice. Exp Mol Med 46: e92. http://doi.org/10.1038/emm.2014.10

[15] Koopman R, Schaart G, Hesselink MK (2001) Optimisation of oil red O staining permits combination with immunofluorescence and automated

quantification of lipids. Histochem Cell Biol 116(1): 63-68. http://doi.org/10.1007/s004180100297
[16] van den Broek MA, Shiri-Sverdlov R, Schreurs JJ, et al. (2013) Liver manipulation during liver surgery in humans is associated with hepatocellular damage and hepatic inflammation. Liver Int 33(4): 633-641. http://doi.org/10.1111/liv.12051
[17] Bezborodkina NN, Chestnova AY, Vorobev ML, Kudryavtsev BN (2016) Glycogen content in hepatocytes is related with their size in normal rat liver but not in cirrhotic one. Cytometry A 89(4): 357-364. http://doi.org/10.1002/cyto.a.22811
[18] Hocher B, Haumann H, Rahnenfuhrer J, et al. (2016) Maternal eNOS deficiency determines a fatty liver phenotype of the offspring in a sex dependent manner. Epigenetics 11(7): 539-552. http://doi.org/10.1080/15592294.2016.1184800
[19] Chaykovska L, von Websky K, Rahnenfuhrer J, et al. (2011) Effects of DPP-4 inhibitors on the heart in a rat model of uremic cardiomyopathy. PLoS One 6(11): e27861. http://doi.org/10.1371/journal.pone.0027861
[20] Schefe JH, Lehmann KE, Buschmann IR, Unger T, Funke-Kaiser H (2006) Quantitative real-time RT-PCR data analysis: current concepts and the novel "gene expression's CT difference" formula. J Mol Med (Berl) 84(11): 901-910. http://doi.org/10.1007/s00109-006-0097-6
[21] Weber M, Davies JJ, Wittig D, et al. (2005) Chromosome-wide and promoter-specific analyses identify sites of differential DNA methylation in normal and transformed human cells. Nat Genet 37(8): 853-862. http://doi.org/10.1038/ng1598
[22] Xia J, Wishart DS (2016) Using MetaboAnalyst 3.0 for Comprehensive Metabolomics Data Analysis. Curr Protoc Bioinformatics 55: 14 10 11-14 10 91. http://doi.org/10.1002/cpbi.11
[23] Chong J, Wishart DS, Xia J (2019) Using MetaboAnalyst 4.0 for Comprehensive and Integrative Metabolomics Data Analysis. Curr Protoc Bioinformatics 68(1): e86. http://doi.org/10.1002/cpbi.86
[24] Miyamoto Y, Mauer AS, Kumar S, Mott JL, Malhi H (2014) Mmu-miR-615-3p regulates lipoapoptosis by inhibiting C/EBP homologous protein. PLoS One 9(10): e109637. http://doi.org/10.1371/journal.pone.0109637
[25] Cui X, Fu J, Luan J, et al. (2020) CircZNF609 is involved in the pathogenesis of focal segmental glomerulosclerosis by sponging miR-615-5p. Biochem Biophys Res Commun 531(3): 341-349. http://doi.org/10.1016/j.bbrc.2020.07.066
[26] Wu B, Guo Y, Chen Q, Xiong Q, Min S (2019) MicroRNA-193a Downregulates HMGB1 to Alleviate Diabetic Neuropathic Pain in a Mouse Model. Neuroimmunomodulation 26(5): 250-257. http://doi.org/10.1159/000503325
[27] Ju B, Nie Y, Yang X, et al. (2019) miR-193a/b-3p relieves hepatic fibrosis and restrains proliferation and activation of hepatic stellate cells. J Cell Mol Med 23(6): 3824-3832. http://doi.org/10.1111/jcmm.14210
[28] Li R, He J, Chen X, et al. (2014) Mmu-miR-193 is involved in embryo implantation in mouse uterus by regulating GRB7 gene expression. Reprod Sci 21(6): 733-742. http://doi.org/10.1177/1933719113512535
[29] Wang X, Li Y, Gong B, Zhang K, Ma Y, Li Y (2021) miR-199b-5p enhances the proliferation of medullary thymic epithelial cells via regulating Wnt signaling by targeting Fzd6. Acta Biochim Biophys Sin (Shanghai) 53(1): 36-45. http://doi.org/10.1093/abbs/gmaa145

[30] Chen T, Margariti A, Kelaini S, et al. (2015) MicroRNA-199b Modulates Vascular Cell Fate During iPS Cell Differentiation by Targeting the Notch Ligand Jagged1 and Enhancing VEGF Signaling. Stem Cells 33(5): 1405-1418. http://doi.org/10.1002/stem.1930
[31] da Costa Martins PA, Salic K, Gladka MM, et al. (2010) MicroRNA-199b targets the nuclear kinase Dyrk1a in an auto-amplification loop promoting calcineurin/NFAT signalling. Nat Cell Biol 12(12): 1220-1227. http://doi.org/10.1038/ncb2126
[32] He Q, Wang F, Honda T, James J, Li J, Redington A (2018) Loss of miR-144 signaling interrupts extracellular matrix remodeling after myocardial infarction leading to worsened cardiac function. Sci Rep 8(1): 16886. http://doi.org/10.1038/s41598-018-35314-6
[33] Qian W, Jin F, Zhao Y, et al. (2020) Downregulation of microRNA-144 inhibits proliferation and promotes the apoptosis of myelodysplastic syndrome cells through the activation of the AKAP12-dependent ERK1/2 signaling pathway. Cell Signal 68: 109493. http://doi.org/10.1016/j.cellsig.2019.109493
[34] He Q, Wang F, Honda T, Greis KD, Redington AN (2020) Ablation of miR-144 increases vimentin expression and atherosclerotic plaque formation. Sci Rep 10(1): 6127. http://doi.org/10.1038/s41598-020-63335-7
[35] Liu Y, Xu J, Gu R, et al. (2020) Circulating exosomal miR-144-3p inhibits the mobilization of endothelial progenitor cells post myocardial infarction via regulating the MMP9 pathway. Aging (Albany NY) 12(16): 16294-16303. http://doi.org/10.18632/aging.103651
[36] Pan Q, Kuang X, Cai S, et al. (2020) miR-132-3p priming enhances the effects of mesenchymal stromal cell-derived exosomes on ameliorating brain ischemic injury. Stem Cell Res Ther 11(1): 260. http://doi.org/10.1186/s13287-020-01761-0
[37] Hu Z, Zhang L, Wang H, et al. (2020) Targeted silencing of miRNA-132-3p expression rescues disuse osteopenia by promoting mesenchymal stem cell osteogenic differentiation and osteogenesis in mice. Stem Cell Res Ther 11(1): 58. http://doi.org/10.1186/s13287-020-1581-6
[38] Kariba Y, Yoshizawa T, Sato Y, Tsuyama T, Araki E, Yamagata K (2020) Brown adipocyte-derived exosomal miR-132-3p suppress hepatic Srebf1 expression and thereby attenuate expression of lipogenic genes. Biochem Biophys Res Commun 530(3): 500-507. http://doi.org/10.1016/j.bbrc.2020.05.090
[39] Mziaut H, Henniger G, Ganss K, et al. (2020) MiR-132 controls pancreatic beta cell proliferation and survival through Pten/Akt/Foxo3 signaling. Mol Metab 31: 150-162. http://doi.org/10.1016/j.molmet.2019.11.012
